# The response strategy and the place strategy in a plus‐maze have different sensitivities to devaluation of expected outcome

**DOI:** 10.1002/hipo.22847

**Published:** 2018-04-23

**Authors:** Yutaka Kosaki, John M. Pearce, Anthony McGregor

**Affiliations:** ^1^ Department of Psychology Durham University Durham DH1 3LE United Kingdom; ^2^ School of Psychology Cardiff University Cardiff CF10 3AT United Kingdom; ^3^ Department of Psychology Waseda University, Shinjuku Tokyo 162‐8644 Japan

**Keywords:** goal‐directed action, hippocampus, place strategy, response strategy, stimulus‐response habit

## Abstract

Previous studies have suggested that spatial navigation can be achieved with at least two distinct learning processes, involving either cognitive map‐like representations of the local environment, referred to as the “place strategy”, or simple stimulus‐response (S‐R) associations, the “response strategy”. A similar distinction between cognitive/behavioral processes has been made in the context of non‐spatial, instrumental conditioning, with the definition of two processes concerning the sensitivity of a given behavior to the expected value of its outcome as well as to the response‐outcome contingency (“goal‐directed action” and “S‐R habit”). Here we investigated whether these two versions of dichotomist definitions of learned behavior, one spatial and the other non‐spatial, correspond to each other in a formal way. Specifically, we assessed the goal‐directed nature of two navigational strategies, using a combination of an outcome devaluation procedure and a spatial probe trial frequently used to dissociate the two navigational strategies. In Experiment 1, rats trained in a dual‐solution T‐maze task were subjected to an extinction probe trial from the opposite start arm, with or without prefeeding‐induced devaluation of the expected outcome. We found that a non‐significant preference for the place strategy in the non‐devalued condition was completely reversed after devaluation, such that significantly more animals displayed the use of the response strategy. The result suggests that the place strategy is sensitive to the expected value of the outcome, while the response strategy is not. In Experiment 2, rats with hippocampal lesions showed significant reliance on the response strategy, regardless of whether the expected outcome was devalued or not. The result thus offers further evidence that the response strategy conforms to the definition of an outcome‐insensitive, habitual form of instrumental behavior. These results together attest a formal correspondence between two types of dual‐process accounts of animal learning and behavior.

## 1 INTRODUCTION

In the early half of the twentieth century the major point of dispute in behavioral psychology was what exactly animals learn when they learn. Early theorists viewed animal learning merely as an association between a stimulus (S) and a subsequent response (R), the strength of which is mechanistically modified, or reinforced, by an event that follows the response (e.g., Hull, 1943; Thorndike, 1911). This simple S–R view was later challenged by a series of findings showing that animals appear to possess detailed expectations about the outcome (O) of an action and act purposively to obtain or avoid that outcome (e.g., Tinklepaugh, 1928; Tolman, 1948; Tolman & Gleitman, 1949; Tolman & Honzik, 1930).

The debate between the behaviorist and cognitivist camps was instrumental in fostering at least two types of dual‐process accounts of learning. On one hand, the debate concerned how animals, in most cases rats, learn to navigate in space, which led to an idea that they can navigate with two different strategies; a place strategy that is based on a “mental map‐like representation” of the absolute spatial position of the goal in relation to various stimuli within the environment (e.g., Tolman, 1948), and a response strategy that relies on the formation of an association between a specific cue from the maze and the animal's own kinesthetic response such as turning in a specific direction (e.g., Hull, 1943; Spence & Lippitt, 1946). Various behavioral techniques have been developed to dissociate the two types of navigation strategies.

In one version of such experiments, a rat is trained initially in a T‐maze discrimination, in which learning both the appropriate bodily response and the spatial position of the goal are effective solutions (i.e., “dual‐solution” T‐maze). The rat is then subjected to a probe trial in which it starts from an arm opposite to that used during the training (Tolman, Ritchie, & Kalish, 1947). A rat that had acquired the original discrimination based on the response strategy would make the same turning response and find itself in the opposite location from the original goal location. By contrast, a rat that had learned the location of the goal during training would show a preference for the arm leading to the same goal location. The accumulated evidence has suggested that both types of learning can occur, depending on experimental variables such as the availability and distinctiveness of cues outside the maze, the use of a correction procedure, and the amount of training (for reviews, see Packard & Goodman, 2013; Restle, 1957).

On the other hand, the same behavioral‐cognitive debate also led to more detailed behavioral analyses of instrumental conditioning, often conducted in testing chambers with lever press as a target instrumental behavior; that is, the analysis of the free operant in a non‐spatial context. Through the use of ingenious behavioral assays such as post‐conditioning outcome devaluation (e.g., Adams & Dickinson, 1981) and contingency degradation (e.g., Hammond, 1980), it was made possible to dissociate and more finely define S‐R/reinforcement learning (the behavior controlled by this S–R process is called “habit”) and the purposive, or goal‐directed, form of instrumental learning that depends on an R–O association (the behavior governed by this process is called “goal‐directed action”; for reviews see Dickinson, 1985, 1994). In the typical outcome devaluation procedure, the value of the reinforcer is first decreased by pairing it with an aversive event such as illness, or by taking advantage of sensory‐specific satiety by pre‐feeding the animal with the reinforcer in a context that does not provide the animal with the opportunity to make the instrumental response. The animal's propensity to perform the instrumental response that had previously produced the now‐devalued outcome is then tested. Crucially, this test takes place *in extinction*. Thus, throughout the devaluation and extinction phases, there is no opportunity for the animals to experience the devalued outcome as a result of the instrumental response. Therefore, the devalued reinforcer has no opportunity to modify the strength of an S–R connection directly, and hence any change in the animal's propensity to perform the instrumental response during the extinction test must be attributed to its use of *expectation* about the current value of the instrumental outcome. If, on the other hand, the instrumental response had been established through an S–R/reinforcement process during training and controlled by the same process during the extinction test, the performance should be insensitive to whether or not the outcome is devalued. The devaluation procedure thus offers a diagnostic tool with which one can assess whether a given instrumental response is an S–R habit or a goal‐directed action. In this paradigm, therefore, the dichotomy is made, not on the basis of animals' spatial dispositions, but based on the sensitivity of a given behavior to the expected value of the outcome; that is, its goal‐directedness.

Given the common historical background in the literature, it is surprising that rather little is known about the relationship between these two types of dual‐process accounts of learning; the two spatial learning strategies and the two instrumental learning processes (e.g., Sage & Knowlton, 2000; de Leonibus et al., 2011, see Section 8 for the details of these studies). In the current study, we aimed to find a formal correspondence between the two types of dual‐process theories, by combining those assays used in each area of research; the opposite start arm test and outcome devaluation. The specific question we asked was whether place‐ and response strategies in the spatial domain formally correspond to goal‐directed and habitual instrumental learning processes, respectively.

In Experiment 1, we trained rats on a dual‐solution T‐maze spatial discrimination and conducted a probe trial from the opposite start arm, before which the value of the food outcome was lowered by the off‐baseline specific satiety procedure. In Experiment 2, we addressed the same question using rats with hippocampal lesions, thereby forcing the animals to rely predominantly on the response strategy to acquire the original discrimination (Packard & McGaugh, 1996).

## 2 EXPERIMENT 1

In Experiment 1, hungry rats were trained on a dual‐solution T‐maze discrimination for a food reinforcer. After reaching the learning criterion and immediately before being tested in a probe trial from the opposite start arm, the rats were prefed with either the reinforcer pellets, to induce a sensory‐specific satiety, or the maintenance diet, to preserve the outcome value while equating the general deprivation level. The rats were given two probe trials on separate days, one under each of the two devaluation conditions, with a retraining session conducted in between. The order of devalued and non‐devalued probe trials was counterbalanced across animals.

## 3 METHOD

### 3.1 Subjects

The subjects were 32 experimentally naïve male Lister Hooded rats purchased from Charles River, UK. They were about 5 months old at the start of the experiment. They weighed on average 476.3 g (*SD *=* *38.7), and were food‐deprived to 85% of their free‐feeding body weight.

### 3.2 Apparatus

**Figure 1:**
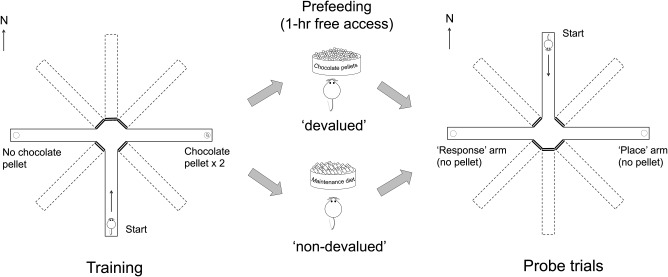
A schematic drawing of the design of Experiment 1. (Left panel) The rats were trained on a dual‐solution T‐maze task in which the start arm and the correct arm were fixed throughout the training. (Middle panel) Immediately preceding the probe trial, rats were given 1‐hr free access to either the reinforcer pellets, for the devalued condition, or the maintenance diet for the non‐devalued condition. (Right panel) The probe trial was conducted in extinction, and the animals were released from the novel arm opposite to the start arm that had been used during training. Each animal received two probe trials, on separate days, one after prefeeding of the reinforcer (devalued probe trial) and the other after prefeeding of the maintenance diet (non‐devalued probe trial). The assignment of the start arm from the four possible arms (N, E, S, and W), the correct arm (left or right), and the order of probe trials (non‐devalued and devalued) were fully counterbalanced across animals

Training and testing took place in an eight‐arm radial maze, which consisted of an octagonal central platform (34‐cm diameter) and eight equally spaced radial arms (87 × 10 cm; Figure 1). The floors of the central platform and the arms were made of wood painted white, while the walls of the arms were made from clear acrylic panels (24‐cm high). At the end of each arm was a circular food well (2 cm in diameter and 0.5 cm deep). At the base of each arm was a transparent Perspex guillotine door (12 cm high) that controlled access to each arm. Each door was operated manually with a string attached to a pulley system. Only three arms, forming a T‐maze, were open and accessible at any given time. Access to each of the remaining arms was blocked by the guillotine door. The entire maze was on a stand (63 cm high) that could be revolved. The maze was installed approximately at the center of a rectangular room (255 × 330 × 260 cm). Illumination was provided by two banks of fluorescent strip lights (0.5 m long, luminance 1022 lux) positioned over the center of the maze. There were various types of visual stimuli around the experimental room, such as posters on the walls, a door, and a small table and a stool close to one wall.

**Figure 2:**
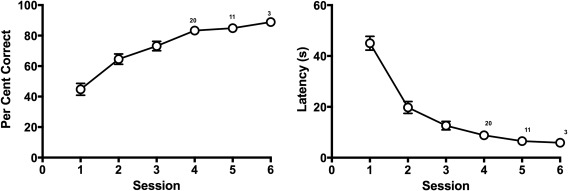
The mean percentage of correct choices (left‐hand panel) and the mean latency to reach a goal location (right‐hand panel) across training sessions in Experiment 1. Note that from Session 4 onwards each session contained a progressively smaller number of animals as more animals reached the criterion. The numbers on the plots on Session 4, 5, and 6 indicate the number of animals that were run on the session

Forty‐five‐mg chocolate‐flavored sucrose pellets (Sandown Scientific, England) were used as reinforcers. Prefeeding occurred in eight identical consumption cages installed in a rack in the holding room. Each cage contained a ceramic ramekin (8 cm diameter and 4 cm deep) that was filled with the chocolate pellets or the maintenance diet, depending on the devaluation condition.

### 3.3 Procedure

#### 3.3.1 Habituation

On the first habituation session, pairs of animals from the same home cage were placed together at the far end of the start arm, and allowed to explore the maze for 10 min. During the first session, a total of 12 pellets were scattered across the entire maze. Three out of eight doors were open to allow access to the start arm and the two choice arms. On the second and third habituation sessions, each animal was run individually for 5 min. Five pellets were placed in each of the two choice arms; three in a food well and two in the alley. If the rat failed to collect all 10 pellets in the third session, additional sessions were run. The start arm was chosen from four arms (N, E, S, and W) for different animals in a counterbalanced way. For each animal, the start arm was consistent throughout the experiment.

#### 3.3.2 Training

Each daily session consisted of eight trials for the first two sessions, and nine trials including one omission trial, inserted at a random position in the trial series, except for the first and the last trials, from Session 3 onward. The omission trial was included in an attempt to make animals' responding more resistant to extinction as we scheduled multiple probe trials.

On each training trial, the rat was placed at the end of the start arm, facing the center of the maze, and allowed to run down the alley and make a choice. The choice was deemed to have occurred once all four legs of the rat were inside the choice arm. If the choice was correct, the rats were able to retrieve two 45‐mg chocolate‐flavored pellets baited in a food well at the end of the arm. The rat was removed from the correct arm 10 sec after finding the reinforcer. If the choice was incorrect, the animals were allowed to stay in the incorrect arm for up to 10 sec before removal, but no track back beyond the choice point was allowed (non‐correction procedure). If the rat attempted a track‐back beyond the choice point to the central octagonal arena, the experimenter picked up the rat and returned it to the holding cage, and recorded an error. No separate correction trial was included. The assignment of the correct arm (left or right) was consistent for each animal throughout the experiment and counterbalanced across animals, orthogonal to the counterbalance of start arm. After an ITI of ∼60 s, the same animal was run on the next trial until it completed all trials in the session. During the ITI, the experimenter wiped clean the start arm and the two goal arms first with 70% ethanol and then wiped them dry with clean paper towel, and re‐baited the correct arm with two chocolate pellets.

#### 3.3.3 Devaluation by prefeeding and probe trial

In order to enable within‐subject comparison, the outcome devaluation was achieved using a prefeeding procedure. Each animal was given two probe trials, one after prefeeding of the chocolate pellets that had been used as a reinforcer during training (devalued probe trial), and the other after prefeeding of the maintenance diet (non‐devalued probe trial). The two probe trials were conducted on separate days, intervened by a retraining session which was run in the same manner as in the original training. During the prefeeding sessions, each animal was individually placed in a consumption cage and allowed 1‐hr free access to one of the food types. Immediately after this prefeeding period, animals were moved to the testing room and run on a probe trial.

In the probe trial, the start arm was opposite to that used during training, and no reinforcer was available at either goal location. If the rat chose an arm which led to the same goal location as that which had been rewarded during training, then the choice was deemed to be based on the “place strategy.” If the rat made the same turning response as that which had been reinforced during training, thereby leading to the location opposite to that which had been rewarded during training, then the choice was deemed to depend upon the “response strategy.”

### 3.4 Learning criterion

A learning criterion was set such that the rat was required to make fewer than four errors across two sessions of training, as well as making the correct response on the first trial on both of the final two sessions. Regardless of the performance, all animals completed at least three sessions of training (25 trials; 2 × eight trials and 1 × nine trials) before being tested in the probe trials. Those animals that failed to reach the criterion within six sessions (52 trials) were removed from the experiment.

## 4 RESULTS

### 4.1 Acquisition

Seven rats that failed to reach the criterion within six sessions of training and one rat that was incorrectly assigned the wrong arm in one training session were removed from subsequent phases of the experiment. Figure 2 shows the acquisition of the T‐maze discrimination from the remaining 24 animals. Note that the data points from Session 4 onward include progressively fewer animals due to attainment of learning criterion. The mean number of sessions required to reach the criterion was 4.42, and the mean number of trials to reach the criterion was 37.75. The mean latency to reach the goal location on the last session in which each animal reached the criterion was 6.33 s (*SD *= 4.26). On the retraining session, which intervened the two probe sessions, the mean latency was 7.15 s (*SD *=* *3.76), which was not statistically different from the last training session for each animal (paired *t* test, *t *=* *0.996, *p *>* *.1). The mean percent correct choice on the retraining session was 85.65, which again was not different from the last training session, 89.35 (paired *t* test, *t *=* *1.556, *p *>* *.1).

### 4.2 Probe trial

**Figure 3:**
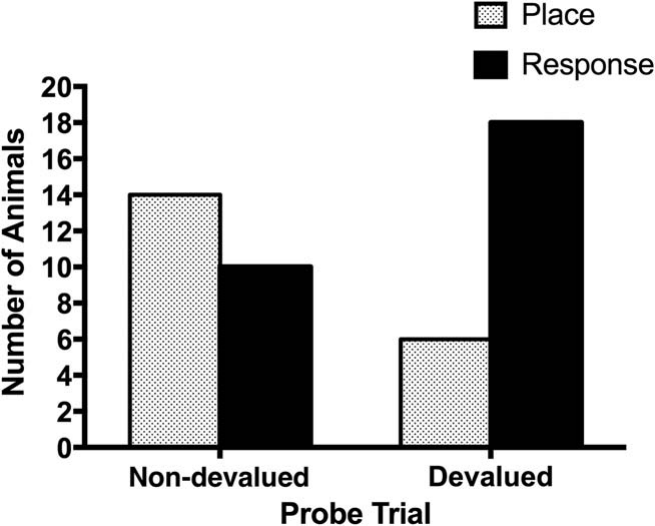
The number of rats that displayed the place strategy (gray bars) and the response strategy (black bars) following the prefeeding of the maintenance diet (non‐devalued probe trial; left) and following the prefeeding of the instrumental reinforcers (devalued probe trial; right)

Figure 3 shows the result from the probe trials. On the non‐devalued probe trial, 14 rats chose the place arm while 10 rats chose the response arm; the difference was not statistically significant (binomial test, *p *>* *.1). By contrast, on the devalued probe trial, six rats chose the place arm while 18 rats chose the response arm; the difference was statistically significant (binomial test, *p *<* *.05). Moreover, a McNemar Test revealed a significant difference in the distribution of animals showing place and response strategies between the devalued and non‐devalued probe trials, *p *<* *.05.

The latency to reach the goal location in the non‐devalued and devalued trials was 9.8 s (*SD *=* *6.22) and 11.7 s (*SD *=* *9.43), respectively. The difference was statistically not significant (paired *t* test, *t *= 0.796, *df *=* *23, *p *>* *.1). Thus, when the expected outcome was devalued, the rats showed a marked preference for the use of response strategy, whereas the animals were indifferent to either strategy when the outcome was not devalued.

As the probe trial was repeated twice for each subject (one non‐devalued trial and the other devalued trial), we investigated whether the repetition of the probe affected the pattern of strategy expression. The results showed that whether the devalued test was conducted first or second did not affect the pattern of strategy expression in the devalued test; of the 12 animals that experienced the devalued trial first, 9 showed the response strategy and 3 showed the place strategy, and the pattern was identical to the other 12 animals for which the devalued test was conducted second (9 response performers and 3 place performers). Similarly, the result of the non‐devalued probe trial was not affected by the order of the test; of the 12 animals that experienced the non‐devalued trial first, 6 showed the place strategy and the other 6 showed the response strategy. Of the other 12 animals (non‐devalued test second), 8 displayed the place strategy and 4 displayed the response strategy.

The fact that the animals did not show a significant reliance on the place strategy in the non‐devalued trial may appear inconsistent with some previous studies which, without involving a devaluation procedure, showed a significant preference for the place strategy early in training (e.g., Packard & McGaugh, 1996). However, as noted in the Introduction, whether animals typically rely on the place or response strategy is affected by various factors, among which is the distinctiveness of the extramaze cues (for a review, see Restle, 1957). Therefore, it is difficult to make an *a priori* assumption about the dominant strategy in a given set of experimental variables. For example, Yin and Knowlton (2004) did not observe a predominance of the place strategy when they tested their rats after just 28 trials of training. It seems likely that in the current experiment, the relatively inconspicuous nature of the extramaze cues, the central illumination that lit the maze evenly from above, and the use of a non‐correction procedure made the task more difficult to solve on the basis of a place strategy, and therefore the animals took longer to acquire the task by recruiting the response strategy to a greater extent than in some previous studies. The argument is also supported by the fact that seven out of 31 rats (22.6%) never reached the learning criterion within six sessions or 52 trials.

The most important finding in the current experiment is that the mild, non‐significant preference for the place strategy in the non‐devalued probe trial was completely reversed if the expected outcome was devalued. There are two possible explanations for such a pattern of results. First, it may be possible that the rats simply tried to avoid the devalued outcome. This account implies that the animals' behavior during the devalued probe trial was controlled by the knowledge about the place of the outcome (i.e., place strategy) *and* the current value of the outcome (i.e., goal‐directed process). That is, the animals did not rely on a response strategy for any part of their behavior in the probe trials. This is unlikely, however, given that the same animals did not show an equally strong preference for the place strategy in the non‐devalued trial. Moreover, we should expect the latency to be longer for such goal‐directed avoidance of the devalued outcome (Sage & Knowlton, 2000), because there is no need for animals to make a choice if not motivated (i.e., paralleling the lower response rates for goal‐directed lever pressing after devaluation; e.g., Adams & Dickinson, 1981), and yet we did not observe a difference in the choice latency.

The second explanation is that the differential expressions of place and response strategies in the non‐devalued and devalued trials reflected different goal‐sensitivities of the two strategies. If it is assumed that the place strategy is inherently sensitive and the response strategy is insensitive to the current value of the expected outcome, then in the probe trial after devaluation the choice should be biased to the one controlled by the goal‐insensitive response strategy, to the extent that the response strategy had been acquired.

## 5 EXPERIMENT 2

The results of Experiment 1 suggested that spatial navigation controlled by the response strategy is insensitive to the current value of the expected outcome, whereas the navigation based on the place strategy comprises a representation of the expected value of the outcome. A prediction that naturally follows is that a spatial behavior governed solely by the response strategy in the first place should show a reduced sensitivity to the outcome devaluation. We conducted Experiment 2 to confirm the prediction.

One way to test that question would be to use a so‐called ‘single‐solution’ maze task, such as a response‐only‐relevant maze task, in which animals are released from different start arms across trials and the food is consistently placed in an arm that bears a consistent angular relation to the start arm across trials (e.g., Tolman, Ritchie, & Kalish, 1946; Chang & Gold, 2003; Gibson & Shettleworth, 2005). A specific problem expected to arise from the use of such a task in the current context is that the response task usually takes longer to acquire as compared to the dual‐solution task or the place‐only‐relevant task, presumably because of the animals' predisposition to initially rely on the place strategy, which interferes with acquisition of the response task (Tolman et al., 1946; Chang & Gold, 2003). As the amount of training is a critical variable which controls the transition from actions to habits (Adams, 1982; Dickinson, Balleine, Watt, Gonzalez, & Boakes, 1995; Killcross & Coutureau, 2003), we could end up confounding the effects of the strategy required for the solution of a task, and the amount of training, on the sensitivities to the outcome value.

Consequently, we adopted an alternative strategy to test the question, by making selective lesions to the hippocampus (HPC) in rats. It is now widely accepted that a functioning HPC is required for the acquisition and expression of the place strategy. For instance, rats with HPC inactivation or lesions rely less on the place strategy and more on alternative strategies during a conflict probe trial (McDonald & White, 1994; Packard & McGaugh, 1996). Hippocampus‐lesioned animals also show impaired acquisition of a place‐only‐relevant version of a plus‐maze task (Chang & Gold, 2003; Compton, 2004), a place‐relevant component of a dual‐solution water maze task (Pearce, Roberts, & Good, 1998; Kosaki, Poulter, Austen, & McGregor, 2015), and a passive place learning in the water maze, which precludes the involvement of a response component (Kosaki, Lin, Horne, Pearce, & Gilroy, 2014). On the other hand, Corbit and Balleine (2000), and Corbit, Ostlund, and Balleine (2002), demonstrated that HPC lesions did not impair rats' sensitivity to the expected value of the outcome. Therefore, it is theoretically possible that a spatial behavior governed by the HPC‐independent response strategy, after HPC lesions, remains sensitive to the expected value of the outcome. On the other hand, if the devaluation treatment did not affect the choice pattern of hippocampal‐lesioned rats during the opposite‐start probe trial, then it would offer further support for the conclusion from Experiment 1 that the response strategy is intrinsically an outcome‐insensitive, habitual form of instrumental behavior.

## 6 METHOD

### 6.1 Subject

The subjects were 22 male Lister Hooded rats, about 5 months old at the start of the current experiment. They weighed on average 476.5 g (*SD *=* *48.21) before surgery. Following the surgery, they were given 2 weeks of recovery before participating in an unrelated spatial learning experiment in a water maze. Approximately three weeks after the completion of the water maze experiment, the animals were subjected to a food‐deprivation schedule, under which their body weight was maintained at 85% of their baseline body weight throughout the experimental period. The animals were naïve to the experimental room, the apparatus, food reinforcement and all other aspects of the current experiment.

### 6.2 Surgery

During the surgery, the rats were anaesthetised with a mixture of isoflurane (1%–5%) and oxygen and placed in a stereotaxic frame (David Kopf Instruments). The incisor bar was set at −3.3 mm. The scalp was incised at the midline to expose the skull. A dental drill was used to remove the skull over the target sites. A 2‐µl Hamilton syringe was used to infuse 63 mM ibotenic acid (Tocris Bioscience, Bristol, UK) dissolved in buffered saline bilaterally into the target region. The infusion was made with an infusion pump at the rate of 0.03 µl/min, and each infusion was followed by a 2‐min diffusion time before the syringe was removed. For the HPC lesions, the coordinates for injections and the volume of each injection followed those described by Jarrard (1989); briefly, the lesion of the whole hippocampus was produced with a total of 28 infusions of ibotenic acid bilaterally. For sham lesions, the skull was removed, the dura was exposed and pierced through with the syringe needle at three points per side, but the syringe was not lowered down into the brain. After the infusions of toxin or the sham procedure were complete, the wound was sutured and the rats were allowed to recover in a warm chamber until conscious. A 10‐ml mixture of glucose and saline was injected subcutaneously after surgery to aid recovery. Buprenorphine (0.012 mg/kg) was injected subcutaneously before and after the surgery for pain relief.

### 6.3 Apparatus

The testing took place in an eight‐arm radial maze that was similarly constructed to that used for Experiment 1 and installed in a different room of similar size. Each arm measured 10 cm wide and 70 cm long. Each part of the maze, including the floor, was made of clear acrylic panels, except for the octagonal central platform (10 cm a side) that was made of wood and painted gray. In between the transparent floor panel and a base panel beneath it, uniform gray paper was inserted so that the color was matched between the floors of the arms and the central octagonal platform. At the end of each arm was a small circular hole (4‐cm diameter), into which a metal cup (5‐cm diameter) could be inserted with its lip hanging on around the perimeter of the hole. The center of the food cup was placed 4.5 cm from the end of each arm. Access to each arm could be blocked by a frosted acrylic panel vertically inserted at the base of each arm, where the arm met the central platform.

The entire maze sat centrally on a rotating round table (180‐cm diameter, 30‐cm high from the floor of the room). The maze floor was raised by 12 cm from the surface of the round base. The maze apparatus was installed in a rectangular room (340 × 300 × 245 cm high), equidistant from the two long walls, but closer to one of the short walls with a 50‐cm gap between the edge of the round base and the near short wall. The maze was lit unevenly with two desk lamps placed in the two corners at the opposite ends of the short wall that was closer to the maze. Each lamp gave illumination towards the corner, not to the maze. Extramaze cues were provided by the two lamps, different posters and cards of different shapes and sizes pasted on the wall, an air purifier installed on the floor close to one wall, which also constantly emitted light through indicator LEDs, a small desk with a TV monitor on it, and a dark blue curtain that was hung from the ceiling to the floor outside the edge of the round table, covering about an eighth of the perimeter of the table. These arrangements were taken in order to increase the control by the extramaze cues and hence by the place strategy, as a pilot experiment using this maze revealed that normal rats did not show a place strategy even with a minimum amount of training when the maze was installed centrally in a larger experimental room and lit brightly and evenly by non‐directional ceiling lights.

### 6.4 Procedure

The procedure was identical to that described for Experiment 1, except for the following detail. The start arm assigned for each animal, consistent throughout training, was chosen from three arms, each separated by 90° (S, E, W; number of animals started from S; HPC: *n *=* *4, Sham: *n *=* *4, E; HPC: *n *=* *4, Sham: *n *=* *3, W; HPC: *n *=* *4, Sham: *n *=* *3).

## 7 RESULT

### 7.1 Histology

**Figure 4:**
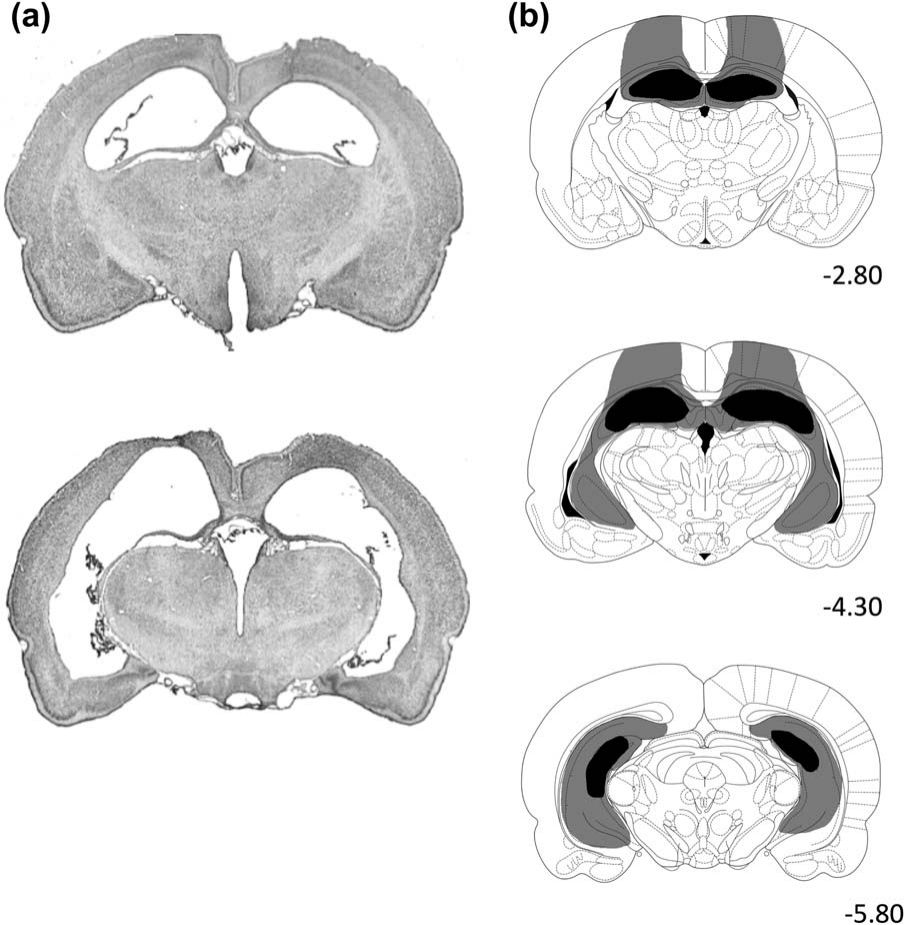
(a) Representative photomicrographs illustrating ibotenic acid lesions of the HPC at the dorsal level (top) and at intermediate and ventral level (bottom). (b) Schematic representations of HPC lesions. The extents of damage from subjects bearing the maximum (gray) and minimum (black) volume of damage are shown. Numbers at the bottom right of each plate represent distance (mm) from Bregma. Atlas plates are from Paxinos and Watson (1998)

Figure 4 shows the reconstruction of HPC lesions. The lesions were almost complete at the septal pole and the dorsal intermediate level, whereas some variations were observed with respect to damage at the ventro‐posterior part. The final number of subjects in each group was as follows; Sham = 10, HPC = 12.

### 7.2 Acquisition

**Figure 5:**
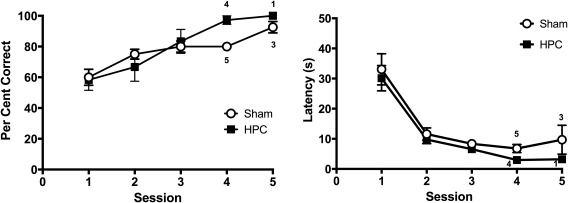
The mean percentages correct choice (left‐hand panel) and the mean latencies to reach a goal location (right‐hand panel) across training sessions in Experiment 2. Note that following Session 3, decreasing number of animals are included in the data plot as animals reached the learning criterion at different points during training, as indicated by the number on each plot. Error bars represent ± *SEM*

Figure 5 shows the result from the acquisition phase. Note that the data points for Session 4 and 5 include progressively fewer animals as more animals reached the criterion. A Lesion × Session ANOVA on the choice data (Figure 5a) during the first three sessions revealed a significant effect of session, *F*(2,62) = 46.42, *p *<* *.001, but the main effect of lesion, *F *<* *1, and Lesion × Session interaction was not significant, *F*(2,40) = 1.62, *p *>* *.1. There was no difference between groups in the mean number of trials to reach criterion; 32.20 and 28.75 for Sham and HPC, respectively (independent *t* test, *t *=* *1.13, *p *>* *.1). A similar analysis on the latency data (Figure 5b) revealed only a significant effect of session, *F*(2,40) = 59.03, *p *<* *.001. There was neither a main effect of lesion nor a Lesion × Session interaction, *F*s* *<* *1.

On the retraining trial, the mean latency to the goal was 8.89 s for Sham and 6.22 s for HPC, which were not different from their respective performance on the last training session (Sham; 8.84 s, *t *=* *0.049, *p *>* *.1, HPC; 5.12 s, *t *=* *0.518, *p *>* *.1). The mean percentage of correct choice on the retraining session was 87.78 for Sham and 98.15 HPC, again not different from those on the last training session: 88.89 for Sham (*t *=* *0.361, *p *>* *.1) and 97.22 for HPC (*t *=* *0.364, *p *>* *.1).

### 7.3 Probe trial

**Figure 6:**
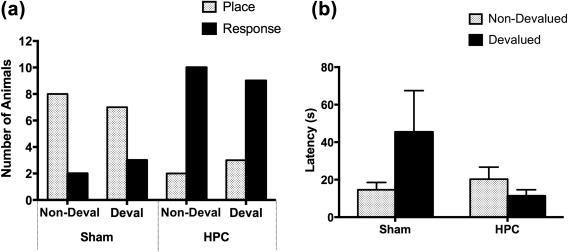
(a) The number of rats that displayed the place strategy (gray bars) and the response strategy (black bars) in probe trials following the prefeeding of the maintenance diet (Non‐Deval) and following the prefeeding of the instrumental reinforcers (Deval) in Experiment 2. (b) The mean latencies to reach one of the goal locations during the non‐devalued and devalued probe trials in Experiment 2. Error bars represent ± *SEM*

The result of primary interest is from the probe trials conducted under the non‐devalued and devalued conditions. The number of animals that displayed the place or the response strategy in each condition is depicted in Figure 6a. The devaluation did not affect the distribution of choices in the sham animals, McNemar test, *p *>* *.1. Thus the data from the non‐devalued and devalued probe trials were combined and subjected to a binomial test, which revealed that Sham rats exhibited an overall preference for the use of the place strategy (binomial test, *p *<* *.05).

The HPC‐lesioned rats, by contrast, showed a substantial preference for the choice that conformed to the response strategy, regardless of whether the reinforcer was devalued or not. A McNemar test revealed no difference in the distribution of place and response choices in the non‐devalued and devalued trials for HPC rats, *p *>* *.1. The data from the two probe trials combined were subjected to a binomial test, which revealed that there were significantly more HPC animals displaying the response strategy than the place strategy, *p *<* *.01.

Although latency to make a choice was not sensitive to devaluation in Experiment 1, it appeared to be so in the current experiment, as latency data during the probe trials demonstrate (Figure 6b). A Lesion × Devaluation ANOVA revealed a marginally significant Lesion x Devaluation interaction, *F*(1,20) = 4.24, *p *=* *.053. Explorations of this trend revealed that Sham rats took significantly longer to reach a goal location in the devalued condition than in the non‐devalued condition, *F*(1,20) = 4.65, *p *<* *.05. The reduction of latencies after devaluation in HPC rats was not significant, *F *<* *1. Thus, while the devaluation did not affect the choice pattern of Sham animals, which was overall in favor of the place strategy, the devaluation did increase the latency of Sham animals but not of HPC animals.

The fact that the devaluation in Sham animals affected the response latency, rather than the choice of arms, in Experiment 2 may appear inconsistent with the result of Experiment 1. The results, however, can be consistently explained by the difference in the extents to which animals learned the response strategy in the two experiments. In Experiment 1, animals developed the response strategy to some extent so that around the half of them showed the response choice in the non‐devalued test. In Experiment 2, however, Sham animals showed a much weaker response strategy and instead demonstrated predominantly the place strategy. It is important to note that whether or not the place strategy predominates at the early training phase depends upon the availability and salience of extramaze cues. In fact, we intended to enhance the normal animals' reliance on the extramaze cues in Experiment 2 based on a pilot experiment conducted in the same room (see Section 6). When the environment in Experiment 2 favored the preferential use of the place strategy as revealed in the choice (unlike in Experiment 1), the devaluation increased the latency to make a choice but did not change the choice. Such a pattern of results in Experiment 1 and Experiment 2 collectively indicates that the devaluation confers the response strategy behavioral control (i.e., animals express the response strategy) *only if* animals had developed the response strategy to some extent, as was the case in Experiment 1. Otherwise, as in Experiment 2, the animals relying only on the place strategy would perform a goal‐directed run with a longer latency, which is analogous to the lower response rates after devaluation in free‐operant experiments. In addition, the increased latency after devaluation is consistent with a previous study that used a win‐shift working memory version of the radial maze task (Sage & Knowlton, 2000; this study will be discussed later). Therefore, the results from the unoperated rats in the two experiments collectively confirm our original prediction that that the response strategy is insensitive to devaluation (habit) and the place strategy is sensitive (action).

Another, and theoretically more important, finding of Experiment 2 was from the HPC rats. First, the HPC‐lesioned rats showed a substantial reliance on the response strategy during the non‐devalued probe trial, as expected from previous findings in a variety of conflict tests in spatial navigation paradigms (Devan & White, 1999; Kosaki et al., 2015; Lee, Duman, & Pittenger, 2008; Mitchell & Hall, 1988; Packard & McGaugh, 1996). Crucially, the HPC animals' reliance on the response strategy was not affected by the outcome devaluation. The latter result thus offers a further support to the conclusion derived from Experiment 1; the response‐strategy‐based navigation formally conforms to the habitual form of instrumental behavior.

## 8 DISCUSSION

The aim of the present study was to seek a formal correspondence between the two types of dual‐process accounts of learned behavior; place versus response strategies in spatial navigation and response‐outcome (action) versus stimulus‐response (habit) processes in instrumental learning.

In Experiment 1, the mild preference for the use of place strategy in a T‐maze under the non‐devalued condition was completely reversed when the expected outcome was devalued, such that the majority of animals displayed the response strategy. The pattern of results indicated that the probe trial performance was concurrently mediated by two processes, the place strategy and the response strategy, and the former was sensitive while the latter was insensitive to the expected value of the outcome. The result therefore suggests that the response strategy meets the formal definition of instrumental habit, whereas the place strategy is a form of goal‐directed action that is sensitive to the expected value of the outcome.

Experiment 2 was conducted in an attempt to answer the same question with one group of animals being forced to rely on the response strategy by means of HPC lesions. On the non‐devalued probe trials, the HPC‐lesioned rats showed predominantly the response strategy as expected, and, crucially, the reliance on the response strategy was unchanged after the outcome devaluation. Again, the result confirms that the response strategy in the spatial domain is a habitual form of instrumental behavior, which is insensitive to the expected goal value.

As previous studies have shown that the HPC is not involved in the representation of the expected value of an instrumental outcome (Corbit & Balleine, 2000; Corbit et al., 2002), the result of Experiment 2 is unlikely to reflect an impairment in the encoding of outcome value *per se*, which would otherwise explain the result independently of the intrinsic associative property of the response strategy. Instead, the result supports the conclusion that the response strategy‐based spatial navigation is inherently insensitive to the goal value, thus meeting the criterion of S–R habit. The conclusion is also congruent with previous findings that both the response strategy in the spatial domain and the instrumental S–R habit in non‐spatial domain depend upon the integrity of the same neural substrate, the dorsolateral striatum (e.g., Packard & McGaugh, 1996; Yin, Knowlton, & Balleine, 2004).

Previously, there have been only a few studies that assessed the issue of outcome representations in spatial navigation with modern behavioral techniques to devalue the reinforcer. Sage and Knowlton (2000) used an eight‐arm radial maze to train rats either on a “win‐stay” S–R version of the task, in which four randomly selected correct arms were signaled by lights on each trial, or on a “win‐shift” working memory version of the task, in which animals needed to remember the four unsignaled baited arms on the first run and to choose the other four arms on the second run. Post‐training devaluation of the food reinforcer did not affect the choice accuracy in either task, but rather increased the choice latency in the win‐shift (working memory) task as well as in the early phase of the win‐stay (reference memory) task. The results indicate that the place‐based working memory performance is a form of goal‐directed behavior, and so is the early‐stage performance in the cue‐approaching win‐stay task, but after extended training cue‐approaching becomes autonomous of goal representations. Thus, the results by Sage & Knowlton are consistent with our current results, despite the difference in nature of the tasks in that our task required animals to make an egocentric left‐right choice rather than an approach to a randomly‐lit arm. Both tasks do not tax allocentric spatial processing, and commonly depend upon the integrity of the dorsal striatum (McDonald & White, 1993; Packard & McGaugh, 1992, 1996).

Given the importance of clarifying the nature of potential interactions between multiple memory systems (e.g., Gibson & Shettleworth, 2005; Poldrack & Packard, 2003), the current results are also interesting as they show that training with more conspicuous place cues somehow make the response strategy underdeveloped. This is a conclusion which is difficult to reach with a standard conflict test without devaluation, as the weak expression of the response strategy in such a test is most likely due to the predominance of the place strategy.

Another study of direct relevance to the current issue of outcome representation in spatial learning was conducted by De Leonibus et al. (2011), in which mice were trained in a dual‐solution T‐maze task just as in the current study. They showed that the choice in a probe trial after overtraining was not affected by outcome devaluation when the mice started from the original start arm, but the devaluation did affect the strategy expression if the animals were tested using the opposite start arm. While the former result is consistent with the current conclusion, the latter appears contradictory. Although a direct comparison of results obtained from different species must be taken with some caution, there appears to be room for explanation for the discrepancy. In the study by De Leonibus et al., the devaluation was achieved by means of conditioned taste aversion across six days and, critically, in parallel with the normal maze training. Such an arrangement effectively allowed the animals to experience the devalued outcome in the goal location a number of times. This raises the possibility that the reduced expression of the response strategy was due to a direct punishment of the S–R habit, rather than reflecting a goal‐directedness of the response strategy. This reduced S–R habit might have been still sufficient to support the animals expressing the response strategy when the stimulus was exactly the same as before on the probe trial with the original start arm, but may have been weakened so as to suffer from generalization decrement when the stimulus to which a response should be made was completely changed except for the intramaze cue, on the probe trial with the opposite start arm. Importantly, when a different group of mice was subjected to even more extensive training on the same task (61 days with 15 trials per day), the response strategy was immune to devaluation regardless of the start arm. The result is thus consistent with the current conclusion, and our results complement their result by showing that place strategy‐based spatial navigation in the dual‐solution T‐maze is sensitive to goal devaluation.

It may require a comment as to the different effects of devaluation for sham animals in Experiments 1 and 2. In Experiment 1, the devaluation resulted in a change in preference from the use of the place strategy to the response strategy while not affecting choice latency. In Experiment 2, the devaluation increased the choice latency while not affecting the choice of arms, which overall indicated the predominance of the place strategy. As already noted in the discussion for Experiment 2, an important difference between Experiment 1 and 2 was that the rats under the non‐devalued condition did not reveal a preference for one strategy over the other in Experiment 1, whereas the sham‐lesioned rats showed a preference for the use of place strategy in Experiment 2. Thus, the devaluation brought about the expression of the response strategy *only if* the animals had acquired the response strategy to some extent in the first place. The different degrees to which the animals acquired the response strategy in the two experiments, then, are most likely to reflect the different availability of extramaze cues; in fact, we intended to enhance the normal animals' reliance on the extramaze cues in Experiment 2 by ways described in the Method section. In other words, with the response strategy underdeveloped in Experiment 2, the animals had no other option but to rely on the place strategy. With the place strategy, navigation came under the control of the currently lowered value of the outcome and therefore the animals performed the run with longer latencies. This is analogous to the low response rate in instrumental lever presses after outcome devaluation (e.g., Adams & Dickinson, 1981; Kosaki & Dickinson, 2010; for a review see Dickinson, 1985).

At a more general level, the current study is one of those attempts to bridge the gap between the two sets of literature in animal learning, one in the spatial domain and the other in the non‐spatial domain. Previous research on this line of approach has successfully demonstrated that spatial learning follows the same learning principles as those demonstrated in non‐spatial associative learning in a number of ways. For example, different associative learning phenomena have been observed in the spatial domain, including overshadowing (e.g., Diez‐Chamizo, Sterio, & Mackintosh, 1985; Horne & Pearce, 2011; Kosaki, Austen, & McGregor, 2013; Redhead, Roberts, Good, & Pearce, 1997), blocking (e.g., Biegler & Morris, 1999; Horne & Pearce, 2009; Roberts & Pearce, 1999), conditioned inhibition (e.g., Horne & Pearce, 2010), latent inhibition (e.g., Chamizo & Mackintosh, 1989), potentiation (e.g., Austen, Kosaki, & McGregor, 2013; Cole, Gibson, Pollack, & Yates, 2011; Pearce, Graham, Good, Jones, & McGregor, 2006), within‐compound associations (e.g., Austen et al., 2013; Austen & McGregor, 2014; Rhodes, Creighton, Killcross, Good, & Honey, 2009), and sensory preconditioning (e.g., Chamizo, Rodrigo, & Mackintosh, 2006; Sawa, Leising, & Blaisdell, 2005). These studies, however, were mainly concerned with the stimulus control aspect of spatial behavior (Pavlovian process), and not profoundly concerned with the instrumental status of the behavior (i.e., S–R or R–O process).

The current conclusion about the instrumental status of place‐ and response‐strategies could offer some account for the apparently inconsistent findings that the above‐mentioned associative phenomena, such as overshadowing and blocking, are not always found, especially when one of the competing cues is provided by the geometry of an arena (e.g., Cheng, 1986; Doeller & Burgess, 2008; Hayward, McGregor, Good, & Pearce, 2003; Kelly, Spetch, & Heth, 1998; McGregor, Horne, Esber, & Pearce, 2009). We have offered at least two, not mutually exclusive, explanations for such an inconsistency (Kosaki et al., 2013; Austen et al., 2013). The present results could serve to further elucidate when spatial learning follows associative rules and when not.

For instance, associative learning principles might apply to spatial navigation only when the training regime makes animals invariably experience single stimulus‐response‐outcome contingency over many repeated trials, as in typical reference‐memory type spatial learning tasks, a condition that favors the development of S–R habits (Holland, 2004; Kosaki & Dickinson, 2010). This may not apply when animals continue to be exposed to multiple stimulus‐response‐outcome contingencies concurrently (as in trial‐unique working memory tasks, with two goal locations, or when testing animals at pre‐asymptotic level of discrimination where animals' choice still retains some variability), a condition that has been shown to keep the behavior under the goal‐directed control even after extended training (Kosaki & Dickinson, 2010). With regard to this issue, it is interesting to note that previous studies have shown that stress can facilitate the use of S–R strategies (Schwabe, Dalm, Schachinger, & Oitzl, 2008; Schwabe, Hoffken, Tegenthoff, & Wolf, 2011; Schwabe & Wolf, 2009; Kim, Lee, Han, & Packard, 2001), and that many of the demonstrations of associative phenomena in the spatial domain, especially where conflicting results exist, were achieved in the water maze, a stressful environment for animals with negative reinforcement as an underlying learning process. Related to this point is a finding by Asem and Holland (2013), who showed that in a submerged plus‐maze in water the rats relied more on the response strategy early in training before switching to the place strategy. Thus, the identification of the precise behavioral process underlying a given spatial behavior is important not only on its own right but also because it merits an attempt to formally relate spatial navigation to non‐spatial learning and behavior, which in turn is critical in fully understanding the neural basis of goal‐directed navigation.

In conclusion, we have demonstrated in two experiments that the two spatial learning strategies, the response strategy and the place strategy, are differentially sensitive to the current value of the expected outcome, and thus each conform to one of the definitions of S–R habit and goal‐directed action, respectively.
